# Mechanical Behavior of Natural Fiber-Based Bi-Directional Corrugated Lattice Sandwich Structure

**DOI:** 10.3390/ma11122578

**Published:** 2018-12-18

**Authors:** Shuguang Li, Yanxia Feng, Mengyuan Wang, Yingcheng Hu

**Affiliations:** Key Laboratory of Bio-based Material Science and Technology of Ministry of Education of China, College of Material Science and Engineering, Northeast Forestry University, Harbin 150040, China; sgliyl@163.com (S.L.); 18845567183@163.com (Y.F.); 18732201721@163.com (M.W.)

**Keywords:** Sandwich structure, Jute fiber, composite material, Bi-directional corrugation

## Abstract

In this study, 11 kinds of composite material were prepared, and the compression behavior of a bi-directional corrugated lattice sandwich structure prepared using jute fiber and epoxy resin was explored. The factors affecting the mechanical behavior of single and double-layer structures were studied separately. The results shows that the fiber angle, length-to-diameter ratio of the struts, and the type of fiber cloth have the most significant influence on the mechanical behavior of the single-layer lattice structure when preparing the core layer. When the fiber angle of the core layer jute/epoxy prepreg is (90/90) the compressive strength and Young’s modulus are 83.3% and 60.0% higher than the fiber angle of (45/45). The configuration of the core and the presence of the intermediate support plate of the double-layer structure have a large influence on the compression performance of the two-layer structure. After the configuration was optimized, the compressive strength and Young’s modulus were increased by 40.0% and 28.9%, respectively. The presence of the intermediate support plate increases the compressive strength, and Young’s modulus of the double-layer structure by 75.0% and 26.6%, respectively. The experimental failure is dominated by the buckling, fracture, and delamination of the core struts.

## 1. Introduction

Lattice sandwich structures have high specific strength and specific stiffness. Therefore, in recent years, the lattice structure of man-made fiber-reinforced composites has been studied extensively [1,2,3,4,5]. Fan et al. used glass fiber to prepare a sandwich structure by the weaving method, and explained its failure mechanism [6], they also studied the failure behavior of multilayer sandwich structures, and concluded that the mechanical properties of multilayer structures can be analyzed from a single-layer [7]. Xiong et al. analyzed the fracture strength and delamination strength of pyramidal lattice core pillars prepared using carbon fiber [8]; double-layer lattice structures with different relative densities were studied, and the multilayer structure was found to have advantages in energy absorption [9]. Hu et al. designed an orthogonal corrugated lattice truss structure that can act as the core to greatly reduce the stress concentration at the nodes [10]. In addition, nylon is used to prepare lattice sandwich structures of different configurations. Studies have shown that honeycomb structures have a greater impact on energy absorption [11]. In honeycomb structures prepared using carbon fiber, the reinforcement provided by the interlocking method is superior to that provided by conventional processing methods, and the configuration is not easily subjected to peel-off [12]. Based on the egg-box core design, carbon fiber, and glass fiber are used to prepare the lattice sandwich structure, and the edge constraint has a great influence on the bearing capacity of the structure [13]. The preparation of the lattice sandwich structure by man-made fibers is lighter and stronger, and the connectivity of the core space is more conducive to the secondary development and application of the core layer structure. These advantages make the lattice sandwich structure have great application prospects. However, raw materials (especially carbon fibers) and complicated preparation processes make it more expensive and difficult to industrialize [14].

The design of materials and structures not only take into account the performance, but also allow for cost control in practical applications [15]. The application of natural fibers is becoming increasingly popular due to its low price, environmental benignity, biodegradability, and good sound insulation [16,17]. When applied to automotive parts, natural fiber-reinforced composites can effectively reduce the energy required for production, and the demand for natural fibers in plastic composites continues to grow at a high rate every year [18]. Alessandra et al. explored the mechanical properties and thermal properties of natural fiber-reinforced composites, and explained their enhancement mechanisms [19]. Petrone et al. used short flax fibers and continuous flax fibers to prepare honeycomb cores, which were recovered by ultrasonic methods, and reported their mechanical behavior under impact tests [20]. Further research also identified two different types of jute/wool felt hybrid composites that are effective in increasing the strength and stiffness of the resultant composite, due to the addition of jute fibers [21]. Composite material reinforced by jute fabric is used to prepare the honeycomb structure, and when loaded, the core layer sustains damage in stable manner and a stress platform appears, unlike the sudden drop of stress that is observed in conventional materials [22]. Jute fiber was used to prepare the honeycomb structure, and three-point bending tests were conducted on specimens of different spans to elucidate their failure modes and mechanisms [23]. Boccarusso et al. used jute fibers to prepare lightweight mesh structures, and demonstrated the desirable impact properties of natural fiber composites [24]. In summary, it can be seen that the noise reduction, degradability and excellent mechanical properties of biomass materials have good application prospects. The use of biomass materials is beneficial for both the cost and the environment. However, existing research on biomass materials are mainly applied to the traditional sandwich structure designs, and the discontinuity of the core space is unfavorable for the development of composite functions.

In this study, a bi-directional corrugated lattice sandwich structure was prepared using jute fiber cloth. Natural fibers are designed to develop lattice sandwich structures that enable secondary design and application of the space left by the core layer. The connectivity of the core space is increased, and the preparation process is more consistent, and the cost is low. Our research combines the advantages of the lattice sandwich structure and the biomass materials. The effects of the fiber angle, the length-to-diameter ratio of the struts, the type of fiber cloth, the fiber volume content, and the configuration of the mechanical properties in compression, were discussed. The effect of the configuration and the presence of an intermediate support plate in the double-layer structure on the compression performance of the entire test piece was also explored. The failure mechanism is discussed and an analytical derivation is made.

## 2. Structure and Materials

### 2.1. Structure and Fabrication

#### 2.1.1. Single-Layer Structure

**Figure 1:**
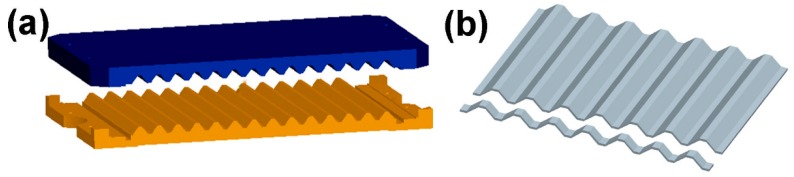
(**a**) Corrugated mold (**b**) Shaped corrugated sheet cut into strips.

The composite materials for the experiment were prepared using hemp fiber cloth (Xitaotao Trading Co., Ltd., Zhejiang, China) and epoxy resin (Nantong Xingchen Synthetic Material Co., Ltd., Nantong, China) without any pretreatment. The first step is the design of a corrugated mold, as shown in Figure 1a. A hemp fiber cloth fully impregnated with an epoxy resin was placed in a mold, and a pressure of 0.3 MPa was applied thereto. The specimen was then cured at room temperature for 48 h. The cured corrugated board is cut into strips (see Figure 1b), periodically cross-combined, and then assembled into a lattice structure using epoxy resin [10].

**Figure 2:**
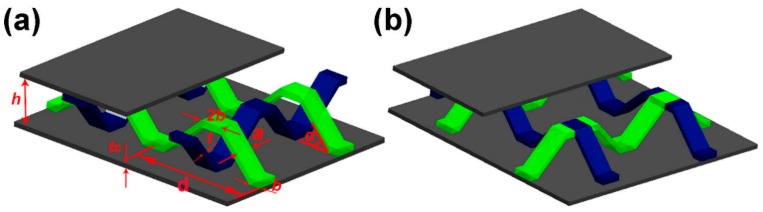
(**a**) Bi-directional corrugated lattice structure (I), (**b**) Pyramidal lattice structure (II).

Two configurations are designed here: the bi-directional corrugation lattice structure (I) shown in Figure 2a, and the pyramidal lattice structure (II), shown in Figure 2b. When the thickness t of the core pillar in the structure is not large, the relative density of the structure, $\bar{\rho }$, can be given by:

(1) $$I:\bar{\rho }=\frac{8(\frac{h-t}{\sin\alpha }+2b)at}{d^{2}h}$$

(2) $$\mathrm{II}:\bar{\rho }=\frac{8(\frac{h-t}{\sin\alpha }+2b)at}{{\left(d+a\right)}^{2}h}$$

*a* (4.8 mm) is the width of the core strut, *b* (2.5 mm) is the strut and panel bonding length, *h* (12 mm) is the core layer height, *t*_c_ (1.5 mm) is the panel thickness, *d* (30 mm) is the total length of a single cell, α (45°) is the angle between the strut and the panel.

#### 2.1.2. Double-Layer Structure

**Figure 3:**
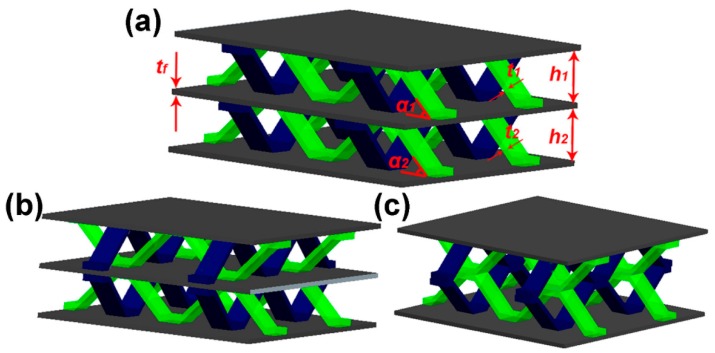
Double-layer structure (**a**) stacked in order (III) (**b**) symmetrically stacked (IV) (**c**) symmetrically stacked but without the intermediate support plate (V).

The double-layer structure is manufactured in the same way as a single-layer structure. Three configurations are designed here: Stacked in order (III), symmetrically stacked (IV), symmetrically stacked, but without the intermediate support plate (V), see Figure 3. The relative density of the structure can be expressed as [9]:

(3) $$\bar{\rho }=\frac{{\bar{\rho }}_{1}h_{1}+{\bar{\rho }}_{2}h_{2}+t_{f}}{h_{1}+h_{2}+t_{f}}$$

Here ${\bar{\rho }}_{1}={\bar{\rho }}_{2}$, so Equation (3) can be expressed as:

(4) $$\bar{\rho }=\frac{2{\bar{\rho }}_{1}h_{1}+t_{f}}{2h_{1}+t_{f}}$$

where ${\bar{\rho }}_{1}$, ${\bar{\rho }}_{2}$, $h_{1}$, $h_{2}$, $\alpha_{1}$, $\alpha_{2}$, $t_{f}$ are identified in Figure 3a.

### 2.2. Materials

**Figure 4:**
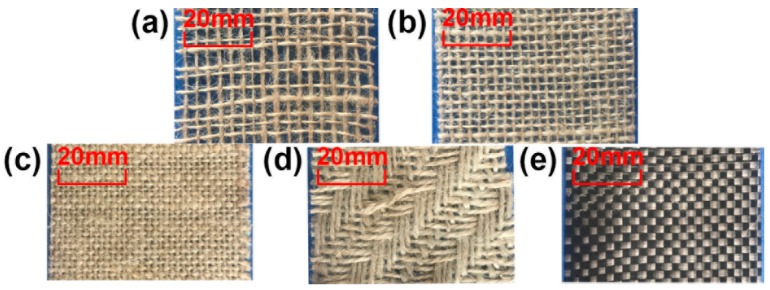
(**a**) Loose weave jute fiber cloth, (**b**) dense jute fiber cloth, (**c**) ultra-dense jute fiber cloth, (**d**) three-line twill jute fiber cloth, (**e**) carbon fiber cloth.

Five materials are used, which are loose-weave jute fiber cloth (A), dense jute fiber cloth (B), ultra-dense jute fiber cloth (C), three-line twill jute fiber cloth (D), and carbon fiber cloth (E), shown in Figure 4. The surface densities of the materials are shown in Table 1.

## 3. Experiment

### 3.1. Core Strut Performance

For this experiment, 11 kinds of composite material were prepared. The compressive strength ($\sigma_{S}$) and Young’s modulus ($E_{S}$) of each composite material were tested by axial compression, and each composite material was tested five times, the results of which are recorded in Table 2.

### 3.2. Out-Of-Plane Compression

**Figure 5:**
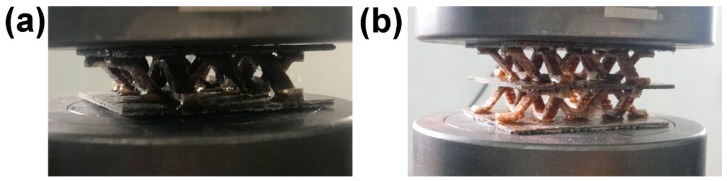
(**a**) Single-layer structure (**b**) Double-layer structure.

The out-of-plane compression test was conducted at a displacement rate of 0.5 mm/min at room temperature with ASTM C365/C 364M-05. There are six sets of comparison experiments in the single-layer structure, and the structure has 2 × 2 cells; The double-layer structure has four configurations to be tested, and the structure has 2 × 2 × 2 cells, shown in Figure 5. The test is repeated five times for each sample. In particular, all of the face sheet are prepared by configuring BEB (According to the angle of the prepreg layer (see Table 2), the composite BEB was prepared by sandwiching a layer of carbon fiber cloth between the two layers of jute cloth.).

The ID of the samples in the experiment are defined. The first half of the ID of the sample refers to the configuration of the sample, and the second half refers to the type of composite material used to prepare the core support of the sample. For example: I in I-CC indicates that the configuration of sample I-CC is a bi-directional corrugated lattice structure (as shown in Figure 2a), CC indicates that the composite material for preparing the core pillar of sample I-CC is the double-layer ultra-dense jute fiber cloth (as shown in Table 2).

## 4. Theoretical Analysis

### 4.1. Single-Layer Structure

**Figure 6:**
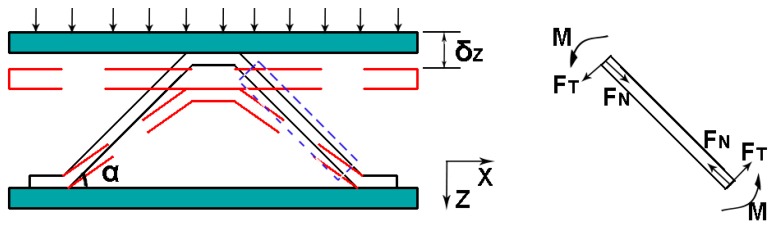
The loading and boundary conditions of the strut in the Z-direction.

For the analysis of a single strut in the core layer, the vertical displacement is $\delta_{z}$, shown in Figure 6, and the axial force $F_{N}$ and tangential force $F_{T}$ can be expressed as [8]:

(5) $$F_{N}=\frac{E_{S}\;at\;\sin^{2}\alpha \delta_{z}}{h-t}$$

(6) $$F_{T}=\frac{E_{S}\;at^{3}\;\sin^{3}\alpha \cos\alpha \delta_{z}}{{\left(h-t\right)}^{3}}$$

where $E_{S}$ is the Young’s modulus of the sandwich strut. Then, the force $F_{z}$ on the single cell in the vertical direction is:

(7) $$F_{z}=4(F_{N}\sin\alpha +F_{T}\cos\alpha )=4F_{N}(\sin\alpha +\frac{F_{T}\cos\alpha }{F_{N}})$$

The stress $\sigma_{Z}$ and strain $\varepsilon_{Z}$ of a single cell can be expressed as:

(8) $$\sigma_{z}=F_{Z}/S$$

(9) $$\varepsilon_{Z}=\delta_{Z}/(h-t)$$

where S is the cross-sectional area of the cell given by $S=\frac{{\left[\frac{2(h-t)}{\cot\alpha }+4b\right]}^{2}}{2}$; then the equivalent compression modulus of a single cell, $E_{Z}$, can be expressed as:

(10) $$E_{Z}=\frac{\sigma_{Z}}{\varepsilon_{Z}}=\frac{8E_{S}\;at\;\sin^{2}\alpha }{{\left[\frac{2(h-t)}{\cot\alpha }+4b\right]}^{2}}[\sin\alpha +\frac{t^{2}\sin\alpha \cos^{2}\alpha }{{\left(h-t\right)}^{2}}]$$

For the axial compression failure, three failure modes are analyzed, namely, Euler buckling of the struts (see Equation (11)), fracture of the struts (see Equation (12)), and delamination failure of the struts (see Equation (13)).

The equivalent compressive strength can be expressed as

(11) $$\sigma_{ZE}=\frac{32\pi^{2}E_{S}I\sin^{2}\alpha }{{\left[\frac{2(h-t)}{\cot\alpha }+4b\right]}^{2}{\left(h-t\right)}^{2}}[\sin\alpha +\frac{t^{2}\sin\alpha \cos^{2}\alpha }{{\left(h-t\right)}^{2}}]$$

Here, the critical Euler buckling load, $F_{E}$, is $F_{E}=\frac{4\pi^{2}E_{S}I\sin^{2}\alpha }{{\left(h-t\right)}^{2}}$ and $I=\frac{at^{3}}{12}$.

(12) $$\sigma_{ZF}=\frac{8\sigma_{CF}at}{{\left[\frac{2(h-t)}{\cot\alpha }+4b\right]}^{2}}[\sin\alpha +\frac{t^{2}\sin\alpha \cos^{2}\alpha }{{\left(h-t\right)}^{2}}]$$

where $\sigma_{CF}$ is the fracture failure stress of the strut.

(13) $$\sigma_{ZD}=\frac{8\sigma_{CD}at}{{\left[\frac{2(h-t)}{\cot\alpha }+4b\right]}^{2}}[\sin\alpha +\frac{t^{2}\sin\alpha \cos^{2}\alpha }{{\left(h-t\right)}^{2}}]$$

$\sigma_{cd}$ is the strength of the struts delamination failure.

### 4.2. Double-Layer Structure

The structural properties of the multilayer composite can be analyzed from a single layer [7]. Therefore, the theoretical analysis of the structure is the same as that for the single-layer structure. The equivalent Young’s modulus of a double-layer structure can be expressed as [9]:

(14) $$E_{Z}=\frac{(h_{1}+h_{2})E_{1}E_{2}}{h_{1}E_{1}+h_{2}E_{2}}$$

Here, *h*_1_ = *h*_2_, and *E*_1_ = *E*_2_. Equation (14) can be expressed as:

(15) $$E_{Z}=E_{i}(i=1\;\mathrm{or}\;2)$$

The failure mode of the double-layer structure is dominated by fracture of the struts, and the equivalent compressive strength can be expressed as:

(16) $$\sigma_{Z}=\sigma_{ZF}$$

## 5. Results and Discussion

### 5.1. Compression of the Single-Layer Structure

**Figure 7:**
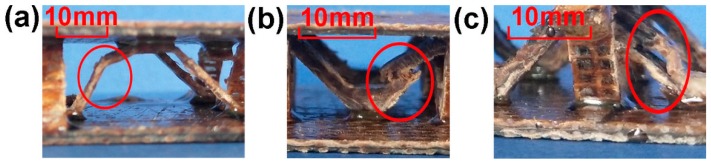
(**a**) Euler buckling of the struts (**b**) Fracture of the struts (**c**) Delamination failure of the struts.

Six sets of experiments were used for comparison. Based on the single-layer structure, we designed the double-layer structure to compare the different mechanical behaviors of the sample when subjected to force loading. This provides a reference for the design of the natural fiber lattice structure from a single-layer structure to a multi-layer structure. The main failure modes pertaining to the experiment are buckling, fracture, and stratification, as shown in Figure 7. All three modes of failure involve a stage of elastic deformation. Buckling refers to the bending and flexing of the core strut with the increase in load, and when buckling reaches the limit, the strut fails.

**Figure 8:**
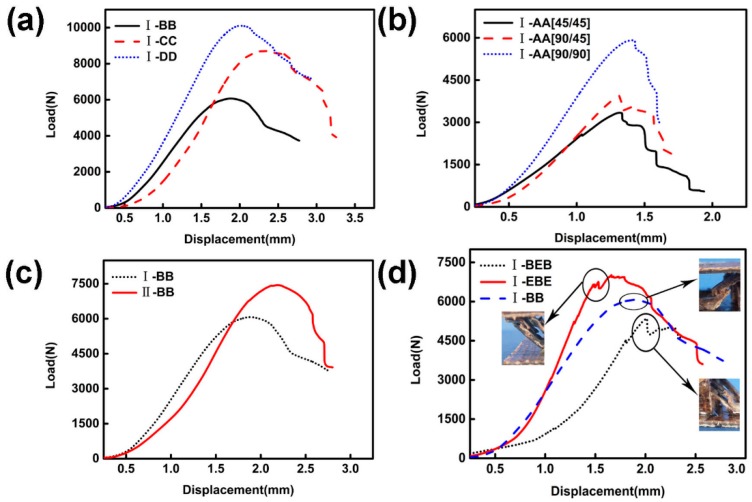
Displacement–load curve for (**a**) different types of jute cloth, (**b**) different fiber angles, (**c**) different configurations, (**d**) different material configurations.

Fracture failure refers to the brittle failure of the core struts, and as the pressure increases, the rupture range of the struts is greater. The damage initiation site of buckling and fracture is mainly determined by the inhomogeneity in the composite [22]. The main failure mode of the struts that incorporate carbon fibers is delamination. Delamination is caused by the failure of the strut interface, and a small range of force drop can be observed on the displacement–load curve diagram, as shown in Figure 8d. The main reason for delamination is the incompatibility between the hydrophilic natural fibers and hydrophobic thermoplastic substrates [18]. The impregnation ability of carbon fiber is limited, and the main binding force arises from the physical combination [25].

#### 5.1.1. Type of Jute Cloth

As shown in Figure 8a, when the relative density of the core layer is 13.6%, two D layers were used to prepare the corrugated lattice structure (I-DD). The compressive strength and Young’s modulus of I-DD are 46.2% and 43.2% higher, respectively, than the corrugated lattice structure (I-BB) prepared using two layers of B. The corrugated lattice structure (I-CC) prepared using two layers of C has a compressive strength of 19.2% higher than that of I-BB, but there is no considerable difference in Young’s modulus. The failure modes of the three samples were all strut fractures.

#### 5.1.2. Fiber Angle

As shown in Figure 8b, a two-layer A was used to prepare a corrugated lattice structure (I-AA). When the core layer was prepared with a fiber layup angle of (90/45), its compressive strength and Young’s modulus were 16.7% and 17.9% higher than those for the layup angle of (45/45), respectively. When the fiber layup angle is (90/90), the compressive strength and Young’s modulus are 83.3% and 60.0% higher than the layup angle of (45/45), respectively. The failure modes of the three specimens were all strut fractures.

#### 5.1.3. Configuration

Two layers of B were prepared with the pyramid structure (II-BB). The compressive strength and Young’s modulus of I-BB were 18.2% and 13.2% higher than II-BB, respectively, as indicated in Figure 8c.

Experiments show that the design of bi-directional corrugated laminates can reduce defects in the fabrication of the structure and the stress concentration [10]. The failure modes of the two specimens were all strut fractures.

#### 5.1.4. Addition of Carbon Fiber Cloth

In order to obtain better composite properties, carbon fiber cloth may be added to the structure. When the relative density of the core layer is 13.6%, the corrugated structure (I-BEB) prepared by adding a layer of carbon fiber cloth has no obvious advantage in terms of the compressive strength and Young’s modulus, relative to I-BB. After adding two layers of carbon fiber cloth (I-EBE), the compressive strength and Young’s modulus of the generated structure were 3.8% and 57.1% higher than I-BB. In addition, the main failure mode of the structure after the addition of carbon fiber cloth is the delamination failure of the struts, as shown in Figure 8d.

#### 5.1.5. Volume Content

**Figure 9:**
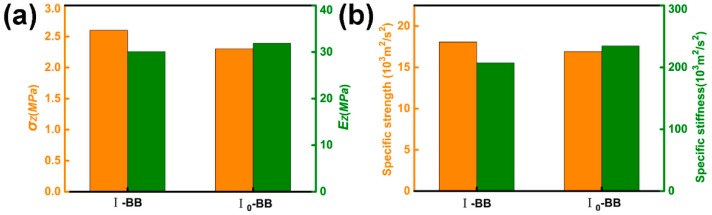
(**a**) Compressive strength and Young’s modulus (**b**) Specific strength and specific stiffness.

Two corrugated lattices I-BB (*t* = 2 mm) and I_0_-BB (*t* = 1.7 mm) were prepared using B. The volume content of hemp fibers in the two struts was 26.5% and 31.2%, respectively (the fiber diameter of B was 0.9 mm). As shown in Figure 9, the Young’s modulus and specific stiffness of I_0_-BB are 6.0% and 13.3% higher than I-BB. The compressive strength and specific strength of I-BB were 14.3% and 7.1% higher than I_0_-BB, respectively.

An increase in fiber volume content is beneficial for an increase in Young’s modulus and specific stiffness, but is detrimental to compressive strength and specific strength.

#### 5.1.6. Length-To-Diameter Ratio of the Struts

**Figure 10:**
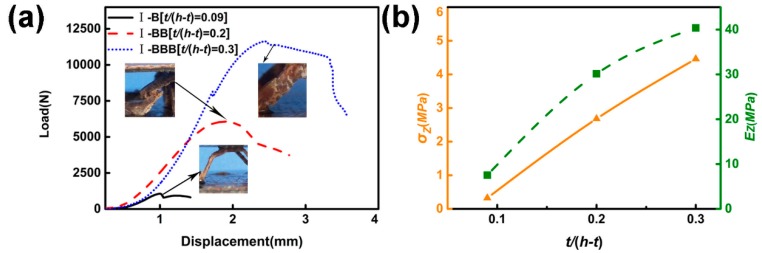
(**a**) Displacement-load curve of I-B, I-BB and I-BBB (**b**) Relationship between compressive strength and *t*/(*h* − *t*).

Three test pieces were prepared by changing the diameter-to-length ratio of the struts (*t*/(*h* − *t*)) of the corrugated lattice structure depending on the number of layers as, respectively, *t*/(*h* − *t*) = 0.1(I-B), *t*/(*h* − *t*) = 0.2 (I-BB), *t*/(*h* − *t*) = 0.3 (I-BBB). As *t*/(*h* − *t*) increases, the rate of increase in strength is linear, while the rate of increase in Young’s modulus becomes smaller, as shown in Figure 10b. As the *t*/(*h* − *t*) changes, the failure mode changes. The failure mode of I-B is buckling, and the failure mode of I-BB and I-BBB is fracture.

**Figure 11:**
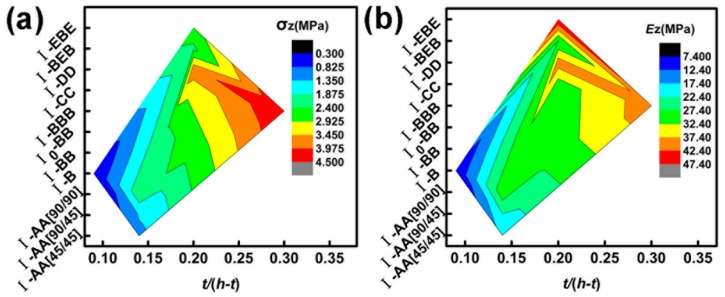
(**a**) Strength–length-to-diameter ratio–configuration comparison diagram (**b**) Young’s modulus–length-to-diameter ratio–configuration comparison diagram.

In summary, changes in the fiber direction and the type of fiber cloth (having a change in surface density, see Table 1) influence the compressive strength and Young’s modulus extensively. The addition of carbon fiber cloth and the increase in the volume content of hemp fiber can effectively improve the Young’s modulus, as shown in Figure 11.

Theoretical analysis and experimental test results are basically consistent, see Table 3.

The theoretical value is greater than the measured value, which may be attributed to structural defects, especially after the addition of carbon fiber. Due to the interfacial interactions between different materials and the matrix, the difference between the theoretical value and the measured value is further enlarged.

### 5.2. Double-Layer Structure

**Figure 12:**
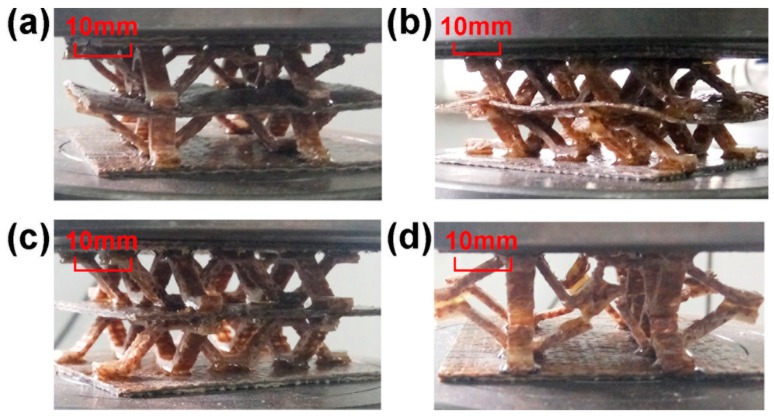
Deformation of test samples under pressure when (**a**) stacked in order (*t_f_*_1_ = 2.0 mm) (**b**) stacked in order (*t_f_*_2_ = 0.9 mm) (**c**) symmetrically stacked (*t_f_*_1_ = 2.0 mm) (**d**) symmetrically stacked but without the intermediate support plate.

**Figure 13:**
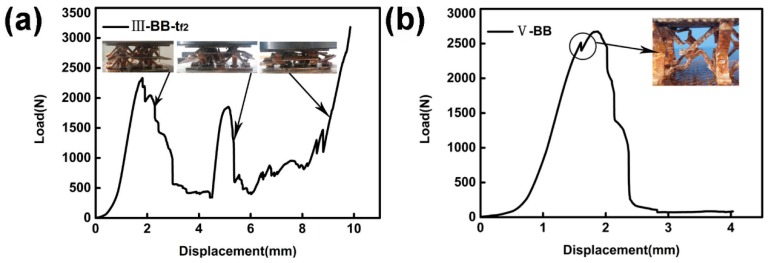
(**a**) The displacement–load diagram of sample III-BB-*t_f_*_2_ (**b**) The displacement–load diagram of sample V-BB.

The core layers of the structure were all prepared using double-layer B and *t* = 1.7 mm. In a double-layer structure with an intermediate support plate, *t_f_*, the test piece deforms as the load increases. The structural configuration of the upper core layer is preferentially destroyed, as shown in Figure 12. The displacement–load curve of the specimen will show double peaks. The damage of each layer in the double-layer structure corresponds to one peak, respectively, until the final test piece is compacted [7]. See Figure 13a for the displacement–load diagram of sample III-BB-*t_f_*_2_. The sample V-BB has no intermediate support plate. When the first strut of the core layer is damaged, it will subsequently damage the other struts connected to it, as shown in Figure 12d. Additionally, there are no double peaks in the displacement–load diagram of V-BB, as shown in Figure 13b.

**Figure 14:**
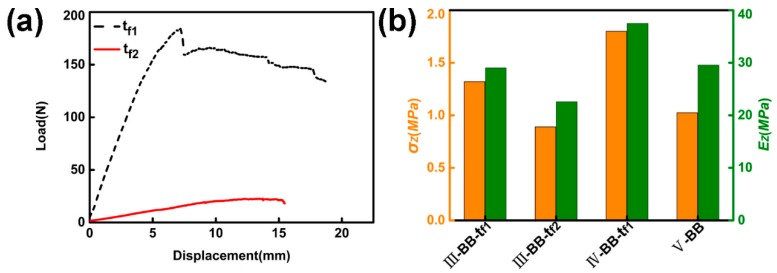
(**a**) Bending displacement–load diagram of the intermediate support plates. (**b**) Compressive strength and Young’s modulus of the double-layer structure.

There are two types of intermediate support plates for the double-layer structure: *t_f_*_1_ = 2.0 mm (prepared by configuring BEB), and *t_f_*_2_ = 0.9mm (prepared by configuring B). The bending strengths are:$\sigma_{tf1}$ = 62.2 MPa, $\sigma_{tf1}$ = 48.7 MPa. The bending displacement–load diagram of the intermediate support plates is shown in Figure 14a. The maximum compressive strength that the sample can withstand prior to failure is indicated in Figure 14b.

The compressive strength and Young’s modulus of III-BB-*t_f_*_1_ were, respectively, 42.9% and 28.2% higher than III-BB-*t_f_*_2_. The configuration was changed to fabricate the type IV-BB-*t_f_*_1_, and the compressive strength and Young’s modulus of IV-BB-*t_f_*_1_ were 40.0% and 28.9% higher than that of III-BB-*t_f_*_1_. The intermediate support plate was removed to prepare V-BB. The compressive strength and Young’s modulus of IV-BB-*t_f_*_1_ were, in turn, 75.0% and 26.6% higher than V-BB.

**Figure 15:**
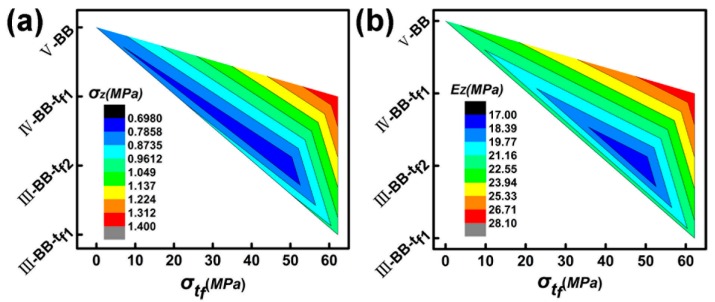
(**a**) Strength–strength of intermediate support plate–configuration comparison diagram. (**b**) Young’s modulus–the strength of intermediate support plate–configuration comparison diagram.

As the strength of the intermediate support plate increases, the compression performance of the test piece also increases. The change in configuration can reduce the dependence of the core layer of the sample on the intermediate support plate, and effectively improve the strength and Young’s modulus of the test piece, as shown in Figure 15. The symmetry of the structure in the double-layer structure is conducive to the stability of the structure, and reduces the dependence on the intermediate support plate. The intermediate support plate also plays a role in stabilizing the upper and lower core layers, and its strength and stiffness affect the mechanical properties of the two-layer structure. As the stiffness and strength increasing, the mechanical properties of the sample increase. This shows that increasing the strength and stiffness of the intermediate support plate and the symmetry of the upper and lower core layers are beneficial to improving the mechanical properties of the sample.

## 6. Conclusions

Eleven kinds of composite material were prepared, and a bi-directional corrugated lattice sandwich structure made of jute fiber was prepared using a mold. The axial compression experiments performed on single-layer and double-layer lattice structures revealed the mechanical properties of the structure, and enabled the analysis of the structural failure mechanism and the proposal of theoretical predictions. The theoretical predictions were then compared with the experimental results, and the following conclusions were drawn:

1. In the sandwich structure prepared by jute fiber cloth and epoxy resin, the mode of core failure is mainly buckling and fracture. Damage initiation in both cases is mainly caused by the inhomogeneity of the fiber composite. The resulting sandwich structure after the addition of carbon fibers mainly fails owing to the delamination failure of the struts. Improving the hydrophilicity of the matrix material and treating the surface of the fiber can contribute to the interfacial bonding ability, thereby improving the mechanical properties.
2. The effects of the fiber angle, type of fiber cloth, fiber volume content, configuration, and length-to-diameter ratio of the struts have an effect on the mechanical properties of the test piece. Optimizing the angle of the jute fiber in the strut so that the orientation of the fiber and axial forces are parallel, helps to significantly increase the compressive strength and Young’s modulus of the sample.
3. In the double-layer lattice sandwich structure, the intermediate support plate acts as a stabilizer between the adjacent layers of the multilayer structure, so that each structural layer has a relatively independent structure. Increasing the strength of the intermediate support plate can effectively prevent the premature instability of the upper core layer, due to the deformation of the support plate, and contribute to the improvement of the compression performance. Optimizing the configuration can reduce the influence of the deformation of the intermediate support plate on the structure, and improve the compressive strength and Young’s modulus.
4. The low-density natural fiber sandwich structure is easy to prepare, and the preparation process is reproducible, highlighting its potential for industrial use as a lattice sandwich structure. Other advantages include its low cost, environmental benignity, and biodegradability.

## Figures and Tables

**Table 1:** Fiber material.

NO.	A	B	C	D	E
Surface density (g/m^2^)	190	220	340	490	210

**Table 2:** Strut parameters.

NO.	*t* (mm)	Material	ω (°)	$\sigma_{S}$ (MPa)	$E_{S}$ (MPa)	$\rho_{S}$ (g/cm^3^)
AA [45/45]	1.4	A	(45/45)	44.1	1234.1	1.09
AA [90/45]	1.4	A	(90/45)	51.6	1496.7	1.09
AA [90/90]	1.4	A	(90/90)	56.7	1509.0	1.09
B	0.9	B	(90)	34.0	896.1	1.10
BB [*t* = 1.7 mm]	1.7	B	(90/90)	61.1	1242.9	1.15
BB [*t* = 2.0 mm]	2.0	B	(90/90)	54.2	989.0	1.07
BBB	3.0	B	(90/90/90)	79.4	1091.5	1.08
CC	2.0	C	(90/90)	64.1	1049.2	1.13
DD	2.0	D	(90/90)	63.8	1377.8	1.20
BEB	2.0	B/E	(90/90/90)	82.9	1835.6	1.22
EBE	2.0	B/E	(90/90/90)	88.0	2733.4	1.02

ω: The angle of prepreg layers $\rho_{S}$: The density of the struts.

**Table 3:** Experimental results and analytical results of a single-layer structure.

NO.	$\bar{\rho }$ (%)	Experimental Results (MPa)		Analytical Results (MPa)	
		$\sigma_{Z}$	$E_{Z}$	$\sigma_{Z}$	$E_{Z}$
I-AA [45/45]	10.0	1.2	19.0	1.9	26.3
I-AA [90/45]	10.0	1.4	22.4	2.2	31.9
I-AA [90/90]	10.0	2.2	30.4	2.4	32.1
I-B	6.6	0.3	7.5	0.3	12.2
I-BB	13.6	2.6	30.1	3.3	30.4
I_0_-BB	11.8	2.3	31.9	3.18	32.3
I-BBB	18.9	4.5	40.4	7.5	51.6
I-CC	13.6	3.1	29.8	4.0	32.3
I-DD	13.6	3.8	40.1	3.9	42.4
I-BEB	13.6	2.3	27.3	5.1	56.4
I-EBE	13.6	2.7	47.3	5.4	84.1
II-BB	11.6	2.2	26.6	3.3	30.4
